# Synergistic targeting of breast cancer stem-like cells by human γδ T cells and CD8^+^ T cells

**DOI:** 10.1038/icb.2017.21

**Published:** 2017-05-09

**Authors:** Hung-Chang Chen, Noémie Joalland, John S Bridgeman, Fouad S Alchami, Ulrich Jarry, Mohd Wajid A Khan, Luke Piggott, Yasmin Shanneik, Jianqiang Li, Marco J Herold, Thomas Herrmann, David A Price, Awen M Gallimore, Richard W Clarkson, Emmanuel Scotet, Bernhard Moser, Matthias Eberl

**Affiliations:** 1 Division of Infection and Immunity, School of Medicine, Cardiff University, Cardiff, UK; 2 INSERM, Unité Mixte de Recherche 892, Centre de Recherche en Cancérologie Nantes Angers, Institut de Recherche en Santé de l’Université de Nantes, Nantes, France; 3 Centre National de la Recherche Scientifique (CNRS), Unité Mixte de Recherche 6299, Nantes, France; 4 Cardiff and Vale University Health Board, University Hospital of Wales, Cardiff, UK; 5 School of Biosciences, Cardiff University, Cardiff, UK; 6 Institute for Virology and Immunobiology, Julius-Maximilians-Universität Würzburg, Würzburg, Germany; 7 Walter and Eliza Hall Institute of Medical Research, Parkville, Victoria, Australia; 8 Systems Immunity Research Institute, Cardiff University, Cardiff, UK; 9 European Cancer Stem Cell Research Institute, Cardiff University, Cardiff, UK

## Abstract

The inherent resistance of cancer stem cells (CSCs) to existing therapies has largely hampered the development of effective treatments for advanced malignancy. To help develop novel immunotherapy approaches that efficiently target CSCs, an experimental model allowing reliable distinction of CSCs and non-CSCs was set up to study their interaction with non-MHC-restricted γδ T cells and antigen-specific CD8^+^ T cells. Stable lines with characteristics of breast CSC-like cells were generated from *ras*-transformed human mammary epithelial (HMLER) cells as confirmed by their CD44^hi^ CD24^lo^ GD2^+^ phenotype, their mesenchymal morphology in culture and their capacity to form mammospheres under non-adherent conditions, as well as their potent tumorigenicity, self-renewal and differentiation in xenografted mice. The resistance of CSC-like cells to γδ T cells could be overcome by inhibition of farnesyl pyrophosphate synthase (FPPS) through pretreatment with zoledronate or with FPPS-targeting short hairpin RNA. γδ T cells induced upregulation of MHC class I and CD54/ICAM-1 on CSC-like cells and thereby increased the susceptibility to antigen-specific killing by CD8^+^ T cells. Alternatively, γδ T-cell responses could be specifically directed against CSC-like cells using the humanised anti-GD2 monoclonal antibody hu14.18K322A. Our findings identify a powerful synergism between MHC-restricted and non-MHC-restricted T cells in the eradication of cancer cells including breast CSCs. Our research suggests that novel immunotherapies may benefit from a two-pronged approach combining γδ T-cell and CD8^+^ T-cell targeting strategies that triggers effective innate-like and tumour-specific adaptive responses.

Cancer stem cells (CSCs) are the principal cause of disease recurrence, distant metastasis, and eventually morbidity and mortality in patients with different malignancies, including breast cancer.^1^ The inherent resistance of CSCs to existing therapies has largely hampered the development of effective treatments for patients with advanced disease, and there is a paucity of studies aiming at directly targeting the CSC pool.^2^ While CSCs are very rare cells and challenging to work with, in particular in humans, progress has been made by linking the cellular epithelial-to-mesenchymal transition (EMT) programme to the generation of CSC-like cells, especially in breast cancer.^3^ In this respect, immortalised human mammary epithelial cells undergoing EMT acquire CSC properties, as judged by their CD44^hi^ CD24^lo^ phenotype, their ability to form mammospheres and their tumour initiation potential.^3, 4, 5^

Immunotherapy offers novel and potentially effective routes to treating cancer, and progress has been made with regard to adoptively transferring expanded or genetically engineered T cells back into patients.^6, 7^ However, the safety and efficacy of CD8^+^ T-cell-based therapies depend on whether the corresponding target antigens are exclusively expressed by tumour cells and not by healthy tissues, and whether they are recognised by the T-cell receptor (TCR) with sufficient affinity. Most importantly, the MHC restriction of tumour-specific epitopes limits the potential benefit of cytotoxic CD8^+^ T cells to patients with appropriate MHC haplotypes.^8^ Alternative immunotherapies are therefore being sought that exploit non-MHC-restricted, ‘unconventional’ T cells that recognise stress-induced changes in transformed cells.^9, 10, 11, 12^ In this context, human Vγ9/Vδ2 T cells have been shown to kill CSC-like tumour initiating cells derived from colon cancer,^13^ ovarian cancer^14^ and neuroblastoma,^15^ especially upon sensitisation of tumour cells by aminobisphosphonates such as zoledronate.

To establish novel immunotherapy approaches that efficiently target CSCs, we here utilised transformed cell lines with CSC-like properties as experimental model for primary breast CSCs, and well-characterised T-cell epitopes as surrogates for yet-to-be-discovered CSC-associated antigens. We demonstrate that the CSC-like cells established in this study are relatively resistant to killing both by antigen-specific CD8^+^ T cell and by Vγ9/Vδ2 T cells. However, the resistance of CSC-like cells to γδ T cells could readily be overcome by inhibition of farnesyl pyrophosphate synthase (FPPS) through pretreatment with zoledronate or with FPPS-targeting short hairpin RNA,^16^ or by opsonisation with the GD2-specific monoclonal antibody hu14.18K322A.^17^ Most importantly, γδ T cells induced upregulation of MHC class I and CD54 on CSC-like cells via secretion of interferon gamma (IFN-γ), and thereby increased the susceptibility to antigen-specific killing by CD8^+^ T cells.

## Results

### Phenotypical characterisation of HMLER-derived CSC-like cells

**Figure 1:**
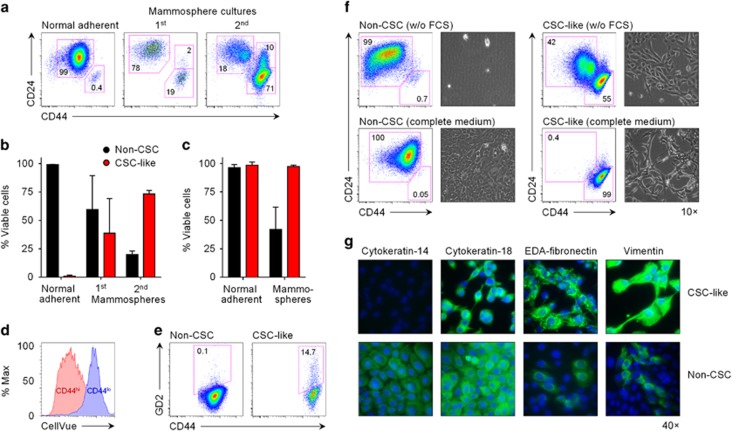
Phenotypical characterisation of HMLER-derived non-CSC and CSC-like cells. (**a**, **b**) Enrichment of CSC-like HMLER cells under mammosphere-forming conditions. HMLER cells from normal adherent cultures or from primary or secondary mammosphere cultures were examined for the proportion of CD44^hi^ CD24^lo^ (CSC-like) cells and CD44^lo^ CD24^hi^ (non-CSC) cells. Gates were set sequentially on intact, single and live cells. Representative fluorescence-activated cell sorting (FACS) plots are shown in (**a**), means±s.d. from three independent cultures in (**b**). (**c**) Differential viability of CSC-like cells and non-CSCs depending on the culture conditions, as assessed by live/dead staining of HMLER cells and gating on intact and single cells. Data shown are means±s.d. from four independent experiments. (**d**) Proliferation of CD44^hi^ cells but not of CD44^lo^ cells in mammosphere cultures of HMLER cells, as assessed by dilution of CellVue labelling (representative of two independent experiments). (**e**) GD2 expression by HMLER cells in normal adherent cultures, gated on CD44^hi^ CD24^lo^ CSC-like cells and CD44^lo^ CD24^hi^ non-CSCs within the parental cell line. FACS plots shown are representative of three independent experiments. (**f**) Stability of CSC-like cells and non-CSCs depending on the culture conditions. FACS-sorted CD44^hi^ CD24^lo^ and CD44^lo^ CD24^hi^ cells were cultured for 14 days in serum-free or complete medium, and examined by flow cytometry and light microscopy. Images shown are representative of two independent experiments. (**g**) Expression of epithelial (cytokeratin-14, cytokeratin-18) and mesenchymal markers (EDA-fibronectin, vimentin) by sorted CSC-like cells and non-CSCs seeded on cover-slip chamber slides and labelled with purified antibodies. AF488-conjugated secondary antibodies were used to visualise stained cells by fluorescence microscopy. Representative images shown were collected from two independent experiments. FCS, foetal calf serum.

We first sought to establish a well-defined cellular model that allows a reliable distinction of CSC-like cells and non-CSCs based on phenotypical, morphological and functional criteria. Immortalised human mammary epithelial cells transformed by overexpression of human telomerase reverse transcriptase, SV40 large T antigen and oncogenic *ras* (referred to as HMLER cells)^18^ showed a predominant CD44^lo^ CD24^hi^ phenotype under adherent culture conditions, yet contained a distinct and stable population of CD44^hi^ CD24^lo^ cells that comprised 0.4–2% of all cells (Figure 1a).^3^ This minor population of putative CSC-like cells could be enriched to >20% of the total population in primary mammosphere cultures, and to >70% in secondary mammosphere cultures (Figures 1a and b), due to drastically reduced survival of CD44^lo^ CD24^hi^ non-CSCs (Figure 1c). At the same time, only CD44^hi^ CD24^lo^ CSC-like cells divided under non-adherent conditions as evidenced by dilution of membrane dyes (Figure 1d). As expected,^4, 19^ antibodies against the ganglioside GD2 stained a proportion of CSC-like cells but not non-CSCs (Figure 1e).

Next, we sorted CD44^hi^ CD24^lo^ CSC-like cells and CD44^lo^ CD24^hi^ non-CSCs from parental HMLER cells to purities >99.5% (Supplementary Figure S1). In complete medium, both cell lines maintained their characteristic phenotype over a period of up to 32 days in adherent culture (Figure 1f, Supplementary Figure S1). Morphologically, non-CSCs displayed an epithelial growth pattern, whereas CSC-like cells had a mesenchymal appearance (Figure 1f), in accordance with the proposed acquisition of CSC properties by cells undergoing EMT.^3^ CSC-like cells stained positively for the mesenchymal markers vimentin and (albeit less prominently) fibronectin extra domain A, whereas only a minor fraction of epithelial-like non-CSCs expressed these markers (Figure 1g). Moreover, CSC-like cells showed no expression of cytokeratin-14 (CK-14) as epithelial marker for the basal/myoepithelial lineage and only intermediate levels of the luminal lineage marker CK-18, as opposed to non-CSCs (Figure 1g). In summary, the phenotype and morphology of CD44^lo^ CD24^hi^ non-CSCs was consistent with epithelial characteristics, while CD44^hi^ CD24^lo^ CSC-like cells showed signs of an incomplete EMT with predominantly mesenchymal characteristics.

### Functional characterisation of HMLER-derived CSC-like cells

**Figure 2:**
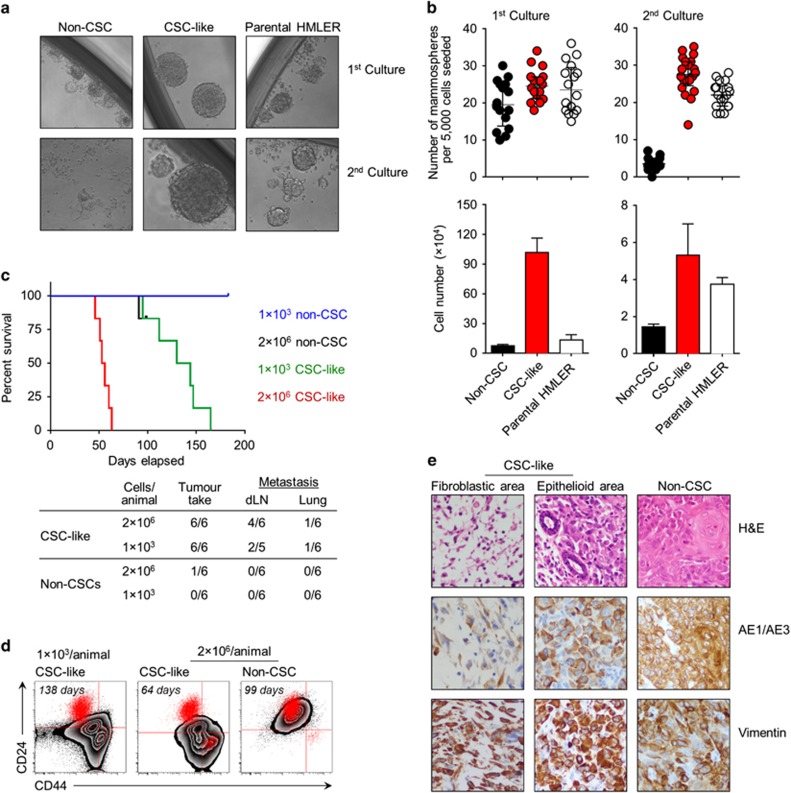
Functional characterisation of HMLER-derived non-CSC and CSC-like cells. (**a**, **b**) Self-renewal under non-adherent conditions. Sorted CSC-like cells and non-CSCs were seeded in ultralow-attachment 96-well plates at a density of 5000 cells per well and cultured in mammosphere medium for 7 days. (**a**) Representative pictures of three independent experiments (× 10 magnification). (**b**) Mammosphere counts and total cell numbers. Each data point represents an independent culture well, error bars depict the median±interquartile range. Data were analysed using one-way ANOVA; asterisks indicate significant differences. (**c**) Tumour take in NSG mice (*n*=6 per group). Mice receiving high doses of CSCs or non-CSCs (2 × 10^6^ cells per animal) were monitored for up to 98 days, and mice receiving low doses (1 × 10^3^ cells per animal) for up to 180 days after injection. End points were determined as no further increase in tdTomato signal over 2 weeks; disease was defined as presence of a palpable tumour with the longest diameter reaching 1 cm. Disease-free survival curves were plotted using the Kaplan–Meier method. The table shows tumour take rate and occurrence of metastasis to lung and draining lymph nodes (dLNs). (**d**) Phenotypical analysis of dissociated tumours derived from injection of FluM1-transduced non-CSCs and CSC-like cells at high and low doses. Tumours were collected when their sizes reached 1000 mm^3^ at the time points indicated. CD44 and CD24 expression of each tumour is shown as zebra plots, with parental HMLER cells as red dots serving as internal reference. FACS plots shown are representative of *n*=6 (left), *n*=6 (middle) and *n*=1 (right) tumours, respectively. (**e**) Histological analysis of collected tumours, shown as H&E staining (top row), and expression of pan-cytokeratin AE1/AE3 (middle row) and vimentin (bottom row). Images are representative sections of *n*=11 CSC-like and *n*=1 non-CSC derived tumours (× 400 magnification). ANOVA, analysis of variance.

In support of their CSC-like phenotype, CD44^hi^ CD24^lo^ cells had a far greater potential to self-renew and form mammospheres than their non-CSC counterparts that formed only very small aggregates (Figure 2a). Moreover, only CSC-like cells but not non-CSCs survived and proliferated under such anchorage-independent culture conditions (Figure 2b). This functional difference was particularly apparent in secondary mammosphere cultures, after dissociation and re-seeding of primary aggregates (Figures 2a and b). The distinct mammosphere-forming abilities of sorted CSC-like cells and non-CSCs replicated both quantitatively and qualitatively the characteristics of the CD44^hi^ CD24^lo^ and CD44^lo^ CD24^hi^ subpopulations, respectively, within the parental HMLER line.

We next determined the tumour take and tumour growth rates of sorted CSC-like cells and non-CSCs in a xenograft model using immunodeficient NOD *scid* gamma (NSG) mice. To this end, we transduced CSC-like cells and non-CSCs with lentiviral vectors that conferred co-expression of the red fluorescent protein tdTomato to allow non-invasive tumour imaging, and of influenza virus matrix protein M1 (FluM1) as surrogate tumour-specific antigen (Supplementary Figure S2). Lentivirally transduced CSC-like cells and non-CSCs were indistinguishable from the corresponding parental cell lines with respect to phenotype, morphology and long-term stability in culture (data not shown). Upon injection into NSG mice, CD44^hi^ CD24^lo^ CSC-like cells showed a striking potential to form tumours in 100% of treated animals, at numbers as low as 1 × 10^3^ CSC-like cells per mouse, as evidenced by *in vivo* imaging of tdTomato fluorescence as well as caliper measurements of palpable tumours (Figure 2c,Supplementary Figure S3). In contrast, CD44^lo^ CD24^hi^ non-CSCs exhibited very poor tumorigenicity with only 1/6 mice developing a sizeable tumour, with much slower growth rate, after receiving 2 × 10^6^ non-CSCs. Fluorescence imaging revealed tumour cells in the lung and draining lymph nodes, but not in non-draining nodes, spleen or liver, of several mice receiving CSC-like cells. No metastasis was observed in mice injected with non-CSCs (Figure 2c).

Finally, we examined the plasticity and differentiation of CSC-like and non-CSCs. In adherent cultures with mammosphere medium, CD44^hi^ CD24^lo^ CSC-like cells expanded and gave rise to CD44^lo^ CD24^hi^ cells with epithelial-like morphology, whereas CD44^lo^ CD24^hi^ non-CSCs failed to survive under such culture conditions (Figure 1f). Tumours derived from CSC-like cells exhibited a capacity to differentiate (Figure 2d), especially after prolonged periods of tumour development (Supplementary Tables S1 and S2). In contrast, tumours derived from non-CSCs showed no signs of differentiation or enrichment of contaminant CSC-like cells (Figure 2d). Histologically, 7/11 tumours arising from CSC-like cells were intimately associated with native mouse mammary ducts, cuffing the vessels with areas of necrosis distal to the vessels. The majority of such tumours showed at least moderate levels of epithelioid differentiation as confirmed by their expression of pan-cytokeratin (AE1/AE3) (Figure 2e); lung metastases showed predominant epithelioid differentiation with no residual features of CSC-like cells (data not shown). However, tumours derived from CSC-like cells uniformly stained for vimentin (Figure 2e), indicative of an only partial reverse EMT process during tumour development *in vivo*. No adenocarcinoma differentiation was identified morphologically, as judged by the absence of carcinoma embryonic antigen expression (Supplementary Table S2).

In summary, HMLER-derived CD44^hi^ CD24^lo^ cells could be maintained stably in culture and manipulated by lentiviral transduction, while displaying phenotypical, morphological and functional features *in vitro* and *in vivo* that are typically associated with breast CSCs. We conclude that such CSC-like cells may represent a powerful experimental model system for the targeting of CSCs, especially CSC subpopulations with EMT-like characteristics, by human immune cells.

### MHC-restricted killing of CSC-like cells by antigen-specific CD8^+^ T cells

CSCs are intrinsically resistant to radiation and chemotherapy, and exploit a number of immune evasion strategies.^2, 20^ To address the recognition of HMLER-derived CSC-like cells and non-CSCs by human T cells, we utilised well-characterised peptides that served as surrogate antigens, namely the immunodominant epitopes of FluM1, p58-66 (GILGFVFTL), and of the human cytomegalovirus (CMV) lower matrix phosphoprotein UL83/pp65, p495-503 (NLVPMVATV). Tumour cells pulsed with FluM1 p58-66 peptides were readily targeted by FluM1-specific CD8^+^ T cells, but not by pp65-specific CD8^+^ T cells as control (Supplementary Figure S4). Similarly, tumour cells pulsed with CMV pp65 p495-503 peptides were only lysed by pp65-specific CD8^+^ T cells but not by FluM1-specific CD8^+^ T cells, demonstrating the specificity of the experimental system. Of note, while epitope-specific CD8^+^ T cells were able to kill both CSC-like cells and non-CSCs when pulsed with the cognate peptides, CSC-like cells were significantly more resistant to killing (Supplementary Figure S4).

**Figure 3:**
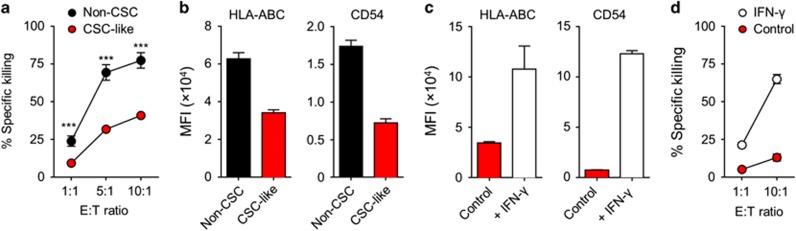
IFN-γ-dependent sensitisation of CSC-like cells to antigen-specific CD8^+^ T cells. (**a**) FluM1-transduced CSC-like cells and non-CSCs were mixed in equal numbers, and used as targets for killing by FluM1-specific CD8^+^ T cells at different effector:target (E:T) ratios. Specific killing of CellVue and PKH67-labelled target cells was assessed by live/dead staining and analysed by flow cytometry. Data shown are from a triplicate experiment representative of two independent experiments. Significance of differences was calculated by two-way ANOVA. (**b**) MHC class I (HLA-ABC) and CD54 expression levels on the cell surface of non-CSCs and CSC-like cells as determined by flow cytometry. Bar diagrams show means+s.d. from three independent experiments. MFI, mean fluorescence intensity. (**c**) MHC class I and CD54 expression levels on CSC-like cells after overnight sensitisation with 100 U ml^−1^ recombinant human IFN-γ as determined by flow cytometry. Results shown are means+s.d. from three independent experiments. (**d**) Sensitisation of FluM1-transduced CSC-like cells to CD8^+^ T-cell-mediated cytotoxicity after overnight sensitisation with 100 U ml^−1^ IFN-γ. Treated and untreated CSC-like cells were mixed in equal numbers, and used as targets for killing by FluM1-specific CD8^+^ T cells at different E:T ratios. Specific killing of CellVue and PKH67-labelled target cells was assessed by live/dead staining and analysed by flow cytometry. Data shown are representative of two experiments performed in triplicate.

Next, we translated these observations to lentivirally transduced target cells that expressed endogenous FluM1. As expected, FluM1^+^ CSC-like cells and FluM1^+^ non-CSCs were both killed by FluM1-specific CD8^+^ T cells. However, CSC-like cells were killed less efficiently than their non-CSC counterparts (Figure 3a). Many tumour cells evade the immune system by downmodulating MHC molecules and other proteins involved in antigen presentation and target cell recognition.^20^ Indeed, HMLER-derived CSC-like cells expressed lower levels of MHC class I and of CD54 (ICAM-1) on the cell surface than non-CSCs (Figure 3b), thereby possibly explaining their relative resistance to CD8^+^ T-cell-mediated killing. Recombinant IFN-γ readily stimulated upregulation of MHC class I and CD54 expression on CSC-like cells (Figure 3c), which in turn led to a significantly improved susceptibility to CD8^+^ T-cell-mediated cytotoxicity (Figure 3d). A similar sensitisation to CD8^+^ T-cell-mediated killing by IFN-γ was observed for non-CSCs (data not shown). These findings demonstrate that IFN-γ effectively sensitises CSC-like cells to killing by tumour antigen-specific T cells.

### Non-MHC-restricted killing of CSCs by innate-like Vγ9/Vδ2 T cells

**Figure 4:**
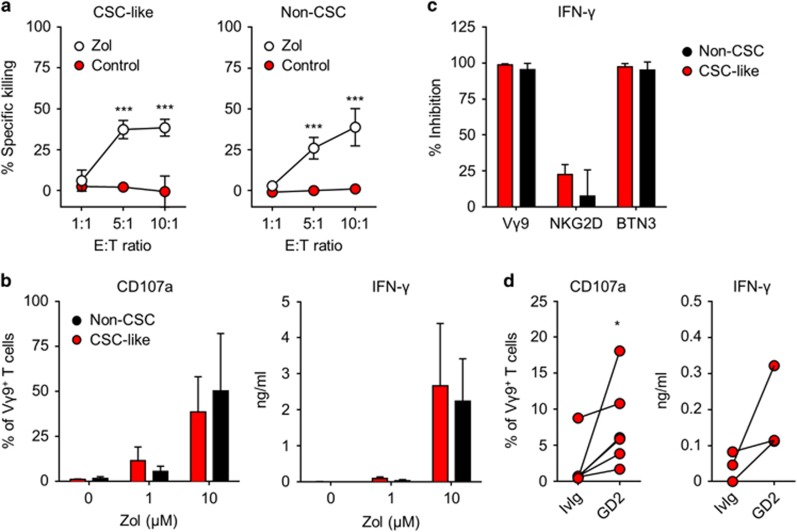
Sensitisation of CSC-like cells to γδ T cells using zoledronate or opsonising antibodies. (**a**) CSC-like cells (left) and non-CSCs (right) treated overnight with 10 μm zoledronate were mixed in equal numbers with untreated CSC-like cells and non-CSCs, respectively, and used as targets for killing by expanded Vγ9/Vδ2 T cells at different effector:target (E:T) ratios. Specific killing of CellVue and PKH26-labelled target cells was assessed by live/dead staining and analysed by flow cytometry. Data shown are from two independent experiments with γδ T cells from three healthy individuals each; differences were assessed by two-way ANOVA. (**b**) γδ T-cell degranulation (left) and IFN-γ secretion (right) in response to CSC-like cells and non-CSCs treated overnight with zoledronate. CD107a mobilisation was measured by flow cytometry in γδ T cells after 5 h of co-culture with target cells in the presence of GolgiSTOP and anti-CD107a; IFN-γ levels were determined after 24 h by ELISA (*n*=4). (**c**) Effect of neutralising antibodies on IFN-γ secretion by γδ T cells in response to CSC-like cells and non-CSCs treated overnight with zoledronate. Data shown are relative inhibition by each blocking antibody as compared with the corresponding isotype controls. Anti-Vγ9 and anti-NKG2D were added directly to target/γδ T-cell co-cultures. For the blocking of BTN3, target cells were incubated with anti-BTN3 for 1 h and then washed before co-culture with γδ T cells. Data shown are means+s.d. from four independent experiments. (**d**) Specific sensitisation of CSC-like cells to γδ T cells by opsonising antibodies. CSC-like cells were co-cultured with expanded γδ T cells in the presence of 10 μg ml^−1^ humanised anti-GD2 monoclonal antibodies or 10 μg ml^−1^ human intravenous immunoglobulin (IvIg) as control. Data show γδ T-cell degranulation (left; *n*=6) and IFN-γ secretion (right; *n*=3) in response to opsonised and control CSC-like cells; differences were assessed by Wilcoxon matched-pairs signed-rank tests.

The dependence of effective tumour cell killing on exogenously provided IFN-γ prompted investigations into the role of γδ T cells, which represent a major and early source of pro-inflammatory cytokines upon activation *in vitro* and *in vivo*.^21, 22^ Human γδ T cells are increasingly appreciated as promising effectors for novel immunotherapy strategies, not the least due to their ability to recognise stress-induced changes in a wide range of transformed cells, including breast cancer cells, in a non-MHC-restricted manner.^11, 12^ Here, both HMLER-derived CSC-like cells and non-CSCs showed a striking resistance to expanded Vγ9/Vδ2 T cells. However, pretreatment of either population with zoledronate resulted in effective activation of co-cultured Vγ9/Vδ2 T cells as judged by targeted cytotoxicity (Figure 4a), as well as mobilisation of CD107a and secretion of IFN-γ (Figure 4b). A similar sensitisation could be achieved via short hairpin RNA-induced knockdown of FPPS, the enzyme inhibited by zoledronate (Supplementary Figure S5).^16^ Confirming the recognition via the TCR, degranulation of Vγ9/Vδ2 T cells and secretion of IFN-γ in response to zoledronate treated CSC-like cells and non-CSCs could readily be blocked by neutralising antibodies against TCR-Vγ9 and butyrophilin 3A (BTN3A/CD277),^23^ but not by antibodies against NKG2D (Figure 4c and data not shown).

Besides recognition via the TCR and NKG2D, Vγ9/Vδ2 T cells have also been shown to target tumour cells including breast cancer cells upon engagement of CD16 (FcγRIII).^24, 25, 26^ In line with the expression of GD2 by CSC-like cells, we observed a relatively modest but detectable enhancement of Vγ9/Vδ2 T-cell responses toward CSC-like cells pretreated with the humanised anti-GD2 antibody hu14.18K322A (Figure 4d). Taken together, these experiments demonstrate that CSC-like cells can be sensitised to recognition by human γδ T cells upon inhibition of FPPS via zoledronate treatment or using short hairpin RNAs, and through the use of CSC-specific opsonising antibodies.

### Synergistic targeting of CSC-like cells by Vγ9/Vδ2 T cells and cytotoxic CD8^+^ T cells

**Figure 5:**
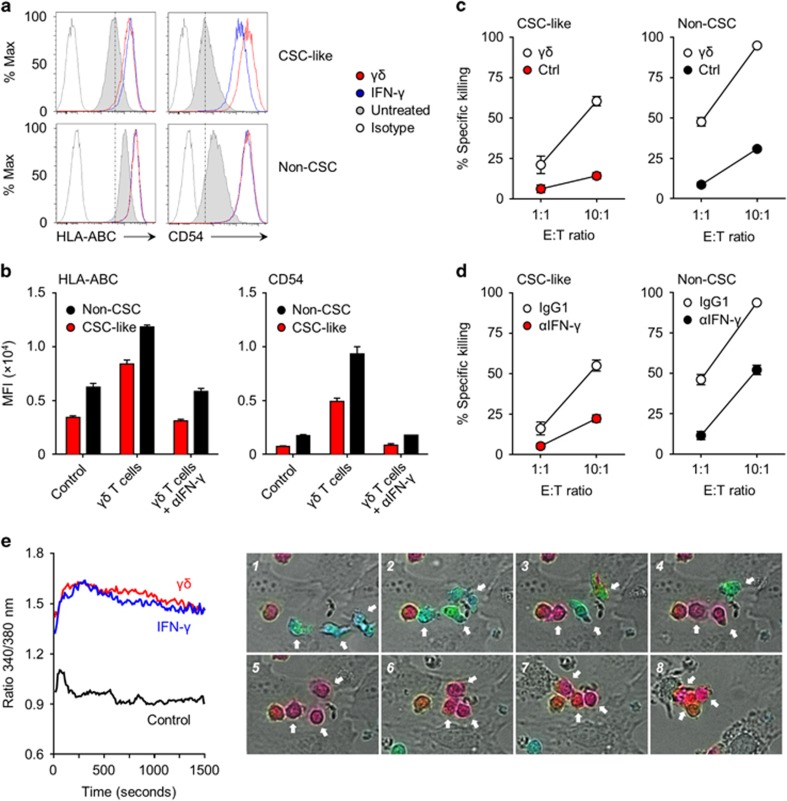
Sensitisation of CSC-like cells and non-CSCs to cytotoxic CD8^+^ T cells by Vγ9/Vδ2 T cells. (**a**) Upregulation of MHC class I (HLA-ABC) and CD54 expression on sorted CSC-like cells and non-CSCs by γδ T cells. Target cells were treated overnight with 1:10 (*v*/*v*) γδ T-cell conditioned medium or with 100 U ml^−1^ recombinant human IFN-γ, and analysed by flow cytometry. Histograms shown are representative for two independent experiments. (**b**) Sorted CSC-like cells and non-CSCs were treated overnight with γδ T-cell conditioned medium in the absence of presence of IFN-γ neutralising antibodies or mouse IgG1 isotype controls, and analysed for their expression of MHC class I (left) and CD54 (right) by flow cytometry. Data shown are representative of two independent experiments using supernatants of expanded γδ T cells from three healthy individuals; differences were assessed by two-way ANOVA. (**c**) Sensitisation of FluM1-transduced CSC-like cells and non-CSCs to CD8^+^ T-cell-mediated cytotoxicity after overnight treatment with 1:10 (*v*/*v*) γδ T-cell conditioned medium. Treated and untreated target cells were mixed in equal numbers, and used as targets for killing by FluM1-specific CD8^+^ T cells at different E:T ratios. Specific killing of CellVue and PKH67-labelled target cells was assessed by live/dead staining and analysed by flow cytometry. Data shown are representative of two independent experiments using supernatants of expanded γδ T cells from three donors. (**d**) Sensitisation of FluM1-transduced CSC-like cells and non-CSCs to CD8^+^ T-cell-mediated cytotoxicity after overnight sensitisation with 1:10 (*v*/*v*) γδ T-cell conditioned medium in the presence of IFN-γ neutralising antibodies or matched isotype controls (IgG1). Treated and untreated target cells were mixed as before, and specific killing was assessed by flow cytometry. Data shown are representative of two independent experiments using supernatants of expanded γδ T cells from three donors. (**e**) Intracellular Ca^2+^ levels as monitored by video microscopy for the indicated acquisition time starting from the moment when Fura-2 AM loaded FluM1-specific CD8^+^ T cells entered in the focal plan. Graphs represent the kinetics of intracellular Ca^2+^ levels, depicted as 340:380 nm ratio; values correspond to the mean emission measured among all T cells present in the field of four independent experiments. Photos are representative pictures of the kinetics of intracellular Ca^2+^ levels and tumour cell killing, using FluM1-transduced CSC-like cells pretreated with γδ T-cell conditioned medium as targets.

Having shown that CSC-like cells can be sensitised to killing by either human αβ T cells and γδ T cells, we addressed the potential synergy of combining the antigen-specific nature of cytotoxic CD8^+^ T cells and the innate killer function of Vγ9/Vδ2 T cells. In line with the general perception that IFN-γ increases tumour immunogenicity,^27^ and with our own observation that recombinant IFN-γ had a striking effect on CSC-like cells (Figure 3), we saw an upregulation of MHC class I and CD54 expression on CSC-like cells upon exposure to supernatants of activated γδ T cells (Figure 5a). By using blocking antibodies, we identified IFN-γ as the main factor in these supernatants (Figure 5b), demonstrating that activated γδ T cells readily boost the potential of CSC-like cells to present antigens to CD8^+^ T cells. A similar γδ T-cell-induced upregulation of MHC class I and CD54 expression was seen with non-CSCs and parental HMLER cells, as well as with a panel of luminal-like and basal-like breast cancer cell lines (MCF-7, SKBR3 and MDA-MD-231) (data not shown), implying that γδ T-cell-derived cytokines generally enhance the susceptibility of breast cancer cells of different origins to be targeted by CD8^+^ T cells.

In support, overnight pretreatment of both FluM1-expressing CSC-like cells and non-CSCs with γδ T-cell-conditioned medium significantly enhanced their susceptibility to killing by FluM1-specific CD8^+^ T cells as compared to untreated controls (Figure 5c). Similarly, γδ T-cell supernatants enhanced the cytotoxic response of FluM1 or CMV pp65-specific CD8^+^ T cells to CSC-like cells and non-CSCs pulsed with the corresponding peptides (data not shown). Blocking with anti-IFN-γ neutralising antibodies diminished the effect of γδ T-cell supernatants on enhancing the cytotoxicity of CD8^+^ T cells toward both CSC-like cells and non-CSCs (Figure 5d).

This γδ T-cell-mediated sensitisation of tumour cells to CD8^+^ T-cell killing was particularly striking when observed in real time using video microscopy, revealing an increased and more persistent calcium flux in CD8^+^ T cells in response to sensitised CSC-like cells (Figure 5e) that resulted in substantial target killing (Figure 5f; Supplementary Movies S1–S3). These findings thus identified non-MHC-restricted γδ T cells as potent adjuvant facilitating subsequent antigen-specific CD8^+^ T-cell immunity against tumour cells, including breast CSC-like cells, through their secretion of IFN-γ.

## Discussion

We identified a powerful synergism between γδ T cell and CD8^+^ T cells in the eradication of tumour cells, including CSC-like cells, suggesting that novel immunotherapies may benefit from a combination of MHC-restricted and non-MHC-restricted approaches. To be able to demonstrate this, we established a stable HMLER-derived cell line with a mesenchymal appearance and a CD44^hi^ CD24^lo^ GD2^+^ phenotype with high expression levels of extra domain A-fibronectin and vimentin. These CSC-like cells readily formed mammospheres under non-adherent conditions, induced subcutaneous tumours in the mammary fat pad of NSG mice at numbers as low as 1 × 10^3^ cells per animal, and had the potential to metastasise to the lung and undergo epithelioid differentiation *in vivo*. We conclude that the present study provides a useful experimental model to study CSC-like cells and non-CSCs derived from the same parental material under identical culture conditions, for a direct comparison of their susceptibility not only to killing by immune cells, but also to chemotherapies and radiation. The stability of HMLER-derived CSC-like and non-CSCs in culture conveniently overcomes the limitations of approaches that depend on long-term sphere cultures, which may change the nature of both CSCs and non-CSCs with respect to differentiation and dedifferentiation. These advantages notwithstanding, the fact that HMLER cells are transformed mammary epithelial cells and not derived from primary breast tumours poses certain limitations, and future work will seek to provide further relevance by sensitising primary CSCs.

Adoptive transfer studies have shown promising potential in patients with different types of tumours, most notably in melanoma.^6, 7, 8^ Currently, such studies are conducted with tumour-infiltrating lymphocytes, chimeric antigen receptors or TCR-engineered T cells. However, all three approaches have relatively limited applicability.^28, 29^ Most importantly, many tumours evade the immune system by downmodulating surface expression of MHC molecules and/or adhesion molecules, especially within the CSC pool.^30, 31, 32, 33^ In agreement, the breast CSC-like cells in the present study expressed relatively low levels of MHC class I and CD54. The poor susceptibility of CSC-like cells to killing by antigen-specific CD8^+^ T cells could be overcome by pretreatment with γδ T-cell conditioned media, demonstrating that γδ T cells are capable of delivering pro-inflammatory cytokines including IFN-γ and rendering poorly immunogenic tumours visible for the immune system. These findings are in accordance with earlier reports showing that IFN-γ rescues MHC class I expression on CSCs of different origins,^31, 32^ and offer hope for efficient targeting of CSCs by adoptively transferred tumour-infiltrating lymphocytes and engineered T cells. However, this study was conducted using well-characterised viral epitopes as surrogate antigens for which high affinity TCRs are available, thereby allowing studies into efficient killing of transduced CSC-like cells by antigen-specific CD8^+^ T cells.^34^ Follow-up experiments therefore need to replicate these findings using relevant tumour-associated antigens, such as aldehyde dehydrogenase 1A1 (ALDH1A1), which was identified as a novel CSC-specific tumour antigen for cytotoxic CD8^+^ T cells in squamous cell carcinoma of head and neck.^35, 36^

The resistance of breast CSC-like cells to γδ T cells could be overcome upon pretreatment with zoledronate, resulting in increased cytotoxicity of γδ T cells. Zoledronate is widely used to prevent excessive bone resorption and skeletal fractures in patients with multiple myeloma, bone metastases and osteoporosis. In addition to its direct effect on the bone, recent meta-analyses provided compelling evidence for a clinical benefit of zoledronate on the development of bone metastases and breast cancer mortality in post-menopausal women or those receiving ovarian suppression therapy.^37^ The underlying mechanisms are unclear, but may stem at least in part from the activity of zoledronate on Vγ9/Vδ2 T cells.^38, 39^ Studies directly aimed at activating Vγ9/Vδ2 T cells in preclinical models and in diverse cancer patient groups have in fact shown promising results, showing that targeting Vγ9/Vδ2 T cells *in vivo* is feasible and safe.^10, 11, 12, 40, 41^ In addition to sensitisation with zoledronate, anti-GD2 antibodies selectively directed Vγ9/Vδ2 T-cell responses against CSC-like cells but not non-CSCs, demonstrating that specific opsonisation represents an alternative approach to sensitise resistant tumour cells to targeted cytotoxicity. Similar strategies have been employed for treating neuroblastoma by natural killer cells,^17, 42^ and for facilitating cross-presentation of tumour antigens by Vγ9/Vδ2 T cells to CD8^+^ T cells.^43^ The relatively weak efficacy of anti-GD2 antibodies may have been due to the variable and often low expression of CD16 on the expanded Vγ9/Vδ2 T cells used in those assays. Besides GD2, further markers with a potential to target Vγ9/Vδ2 T cells specifically against breast CSCs include the human epidermal growth factor receptor 2 (HER2).^44^ Indeed, the HER2-specific monoclonal antibody trastuzumab was recently shown to opsonise human breast cancer xenografts and enhance the ability of γδ T cells to control tumour progression.^26^ The availability of approved drugs and biologics to enhance the TCR-mediated and antibody-dependent cytotoxicity of γδ T cells therefore allows a rapid translation of the present findings in the clinic.

Taken together, we have identified a powerful synergism between MHC-restricted and non-MHC-restricted T cells in the targeting of breast CSC-like cells. Our research provides proof of principle that novel immunotherapies may benefit significantly from combining targeted strategies that trigger effective innate and adaptive responses.^45^ In addition to their cytotoxic effector functions against malignant cells and their ability to boost adaptive αβ T-cell responses by modulating the immunogenicity of transformed cells, human Vγ9/Vδ2 T cells also possess a unique ability to act as professional antigen-presenting cells, including the capacity to cross-present exogenous antigens to CD8^+^ T cells.^43, 46, 47, 48^ These observations lend further credence for the potential of a combined immunotherapy approach where patients receiving autologous tumour-infiltrating lymphocytes or engineered T cells may benefit from a co-administration of *ex vivo* expanded γδ T cells or by concomitant treatment with safe and effective γδ T-cell stimuli such as zoledronate. Such therapy regimes that boost the efficacy of adoptive CD8^+^ T-cell transfer can now be tested in preclinical models and in patients.

## Methods

### Tumour cells

HMLER cells were kindly provided by Dr Robert Weinberg (Whitehead Institute for Biomedical Research, Cambridge, MA, USA) and cultured in DMEM:F12 (1:1) medium (Invitrogen, Paisley, UK) supplemented with 10% foetal calf serum, 10 ng ml^−1^ recombinant human epidermal growth factor (Peprotech, London, UK), 10 μg ml^−1^ insulin (Sigma-Aldrich, Dorset, UK), 0.5 μg ml^−1^ hydrocortisone (Sigma-Aldrich), 1 μg ml^−1^ puromycin (Sigma-Aldrich) and 50 μg ml^−1^ penicillin/streptomycin (Invitrogen).^3, 18^ CD44^hi^ CD24^lo^ CSC-like and CD44^lo^ CD24^hi^ non-CSC-like HMLER cells were sorted to >99.5% purity using a BD FACSAria II and maintained in culture in complete DMEM:F12 medium. The human breast cancer cell lines MDA-MB-231, MCF-7, SKBR3 and BT-474 were cultured using RPMI-1640 medium supplemented with 10% foetal calf serum, 2 mm l-glutamine, 50 μg ml^−1^ penicillin/streptomycin and 100 μm non-essential amino acids (Invitrogen). Mammospheres were grown in ultralow-attachment plates (Corning, Schiphol, Netherlands), using serum-free MEBM medium (Lonza, Slough, UK) supplemented with B27 (Invitrogen), 20 ng ml^−1^ epidermal growth factor (Peprotech), 5 μg ml^−1^ insulin, 0.1 μm β-mercaptoethanol and 1 μg ml^−1^ hydrocortisone (all from Sigma-Aldrich).^49^

### T cells

Human T cells were cultured in RPMI-1640 medium supplemented with 10% foetal calf serum, 2 mm l-glutamine, 1% sodium pyruvate and 50 μg ml^−1^ penicillin/streptomycin. Vγ9/Vδ2 T cells were expanded from peripheral blood mononuclear cells of healthy donors with 1 μm zoledronate (Zometa; Novartis, Basel, Switzerland) and 100 U ml^−1^ IL-2 (Proleukin; Novartis) for 14 days, and further enriched to purities >98% CD3^+^ Vγ9^+^ by negative selection using a modified human γδ T-cell isolation kit that depletes B cells, αβ T cells, NK cells, dendritic cells, stem cells, granulocytes and monocytes (Stem Cell Technologies, Cambridge, UK). Resulting Vγ9/Vδ2 T-cell populations were predominantly CD45RA^−^ CD27^−^ effector/memory cells, with <15% CD45RA^−^ CD27^+^ central memory cells and <5% CD45RA^+^ CD27^−^ terminally differentiated cells; CD16 expression on expanded Vγ9/Vδ2 T cells varied from 6 to 74% CD16^+^ (data not shown). γδ T-cell conditioned medium was generated by culturing purified Vγ9/Vδ2 T cells overnight in the presence of 10 nm HMB-PP (kindly provided by Dr Hassan Jomaa, Justus-Liebig University Giessen, Germany). FluM1-specific and CMV pp65-specific CD8^+^ T cells were expanded from peripheral blood mononuclear cells of HLA-A2^+^ donors to >99% tetramer positivity using the immunodominant peptides of influenza matrix protein, FluM1 p58-66 (GILGFVFTL) and of CMV lower matrix phosphoprotein, UL83/pp65 p495-503 (NLVPMVATV), respectively, at a concentration of 0.1 μm in the presence of 100 U ml^−1^ IL-2 and 20 ng ml^−1^ IL-15 (Miltenyi, Bisley, UK).^47, 48^

### Generation of FluM1^+^ tdTomato^+^ target cells

The cDNA of FluM1 of influenza strain A/Puerto Rico/8/34 (H1N1) was cloned from pMA_MPT_matrx_protein (kindly provided by Dr Mai Ping Tan, Cardiff University) between the *Sal*I and *Xmaj*I cloning sites of the lentiviral transfer vector pELNSxv (kindly provided by Dr James Riley, University of Pennsylvania, PA, USA). PCR reactions were carried out using the Phusion High-Fidelity PCR kit (New England Biolabs, Hitchin, UK) and customised primers; forward, 5′-GAATCCCGGCCCTAGGATGAGCCTGCTGACCGAGGT-3′ reverse, 5′-GAGGTTGATTGTCGACTCACTTGAACCGCTGCATCT-3′ (Eurofins, Wolverhampton, UK). For the production of lentiviral particles containing pELNSxv-tdTomato-T2A-FluM1 vectors, HEK 293 T cells were transiently transfected with lentiviral packaging, envelop and transfer plasmids by CaCl_2_ precipitation. Lentiviral particles were collected and purified for transfection of CSC-like cells and non-CSCs in the presence of 4 μg ml^−1^ polybrene (Sigma-Aldrich). Lentivirally transduced cells were identified based on their expression of tdTomato, and sorted to >98% purity using a BD FACSAria II.

### Constitutive and inducible knockdown of FPPS

Constitutive FPPS knockdown cells were generated as described.^16^ The inducible vector FUTG(INSR), which contains a knockdown construct for rat insulin receptor,^50^ served as negative control for the inducible FPPS knockdown vector SR22 for the target sequence V2HS_228248 (Thermo Scientific, Open Biosystems, Huntsville, AL, USA).^16^ Specific oligos were annealed and subsequently ligated into the *Bbs*I and *Xho*I sites of pH1tet-flex; 5′-TCCCACCAGCAGTGTTCTTGCAATATTTCAAGAGAATATTGCAAGAACACTGCTGGTTTTTTC-3′ (forward) and 5′-TCGAGAAAAAACCAGCAGTGTTCTTGCAATATTCTCTTGAAATATTGCAAGAACACTGCTGGT-3′ (reverse). The H1tet-shRNA22 cassette was cloned into the *Pac*I site of the lentiviral vector FH1tUTG^50^ using specific primers; 5′-CGTGTATTAATTAACCATGGAATTCGAACGCTGAC-3′ (forward) and 5′-CGATCTTAATTAACAGGCTAGCCTAGGACGCG-3′ (reverse). All retroviral or lentiviral constructs were transduced into the respective target cells by transient transfection of HEK 293 T cells using CaCl_2_ precipitation. After 24 h, 10 nm sodium butyrate was added, and virus containing supernatants were collected on the following day and added to target cells in the presence of polybrene.

### Flow cytometry

Cells were acquired on an eight-colour FACSCanto II (BD Biosciences, Oxford, UK) and analysed with FlowJo (TreeStar, Ashland, OR, USA). Single cells of interest were gated based on their appearance in side and forward scatter area/height, exclusion of live/dead staining (fixable Aqua; Invitrogen) and surface staining. The following monoclonal antibodies (mAbs) were used for surface labelling: anti-CD3 (UCHT1), CD8 (HIT8a and SK1), CD16 (3G8), CD24 (ML5), CD44 (G44-26), GD2 (14.G2a) from BD Biosciences; anti-TCR-Vγ9 (Immu360) from Beckman Coulter, High Wycombe, UK; and anti-HLA-ABC (w6/32) from Biolegend, London, UK; together with appropriate isotype controls. Intracellular cytokines were detected using anti-IFN-γ mAbs (B27, BD Biosciences). Surface mobilisation of CD107a was detected by adding anti-CD107a (H4A3; BD Biosciences) mAbs and GolgiStop (BD Biosciences) to cultures for 5 h prior to flow cytometric analysis.

### Functional T-cell assays

CD107a mobilisation, expression of activation markers and cytokine production were assessed by flow cytometry-based assays as described previously for the activation of γδ T cells and CD8^+^ T cells.^48^ γδ T cells and CD8^+^ T cells treated with PMA and ionomycin were used as positive control in functional assays. For the sensitisation, CSC-like cells and non-CSCs were treated overnight with 10 μm zoledronate (Zometa; Novartis), washed and used as targets in co-culture with effector T cells at specified effector:target (E:T) ratio. Cytotoxicity assays were conducted in co-cultures of two distinct target cell populations to assess preferential killing of specific targets.^48, 49^ In brief, two different target cell populations were labelled separately with different lipophilic dyes (PKH26, PKH67 or CellVue; all from Sigma-Aldrich), and mixed at 1:1 ratio for subsequent co-culture with effector T cells at different E:T ratios. After 4 h at 37 °C, cultures were collected, stained using the Live/dead fixable Aqua dead cell stain kit (Invitrogen) and acquired on a BD FACSCanto II. The analysis was performed by serial gating on single cells (FSC-A/FSC-H) and distinctively stained targets (for example, CellVue^+^ PKH67^−^ or CellVue^−^ PKH67^+^), and the proportion of dead cells was determined for each target population. In these functional assays, the neutralising antibodies anti-TCR-Vγ9 (Immu360; Beckman Coulter), anti-BTN3A (103.2; Dr Daniel Olive, Institut Paoli Calmettes, Marseille, France), anti-NKG2D (1D11; Biolegend) and anti-IFN-γ (B27; Biolegend) were used at 10 μg ml^−1^. To test the role of opsonising antibodies, tumour cells were pretreated with anti-GD2 (hu14.18K322A; Dr Fariba Navid, St Jude Children’s Research Hospital, Memphis, TN, USA) for 30 min at 10 μg ml^−1^. Levels of secreted IFN-γ in culture supernatants were determined by ELISA (Biolegend, eBioscience, Cheshire, UK), using a Dynex MRX II reader.

### Animal studies

All procedures were performed in accordance with the Animals (Scientific Procedures) Act 1986 and approved by the UK Home Office under project license 30/2891. Surgery was performed under isoflurane anaesthesia, and every effort was made to minimise suffering. Female NSG mice were purchased from Charles River Laboratories at 5–7 weeks of age and housed in specific pathogen‐free conditions. For xenograft transplantations, the indicated numbers of tumour cells (ranging from 1 × 10^3^ to 2 × 10^6^ per mouse) were resuspended in a mixture of DMEM/F12 medium with Matrigel (Corning) at 1:1 ratio and injected s.c. in NSG mice near the mammary fat pad. Tumour growth was monitored twice a week by external measurement of xenografts using a Vernier caliper and by fluorescence imaging (Kodak FX-PRO, Rochester, NY, USA). Mice were culled before tumours reached 1.5 cm in diameter. For flow cytometric analyses or cell sorting, tumours were excised, chopped into pieces using scalpel blades and mashed with a syringe plunger. The resulting cell suspension was passed through 70 μm nylon cell strainers (BD Falcon) and stained with indicated panel of mAbs. Tumours, tumour‐draining inguinal lymph nodes and the contralateral non-draining lymph nodes as well as livers, lungs, brains and spleens were collected for fluorescence imaging, and subsequently fixed in neutral buffered formalin and embedded in paraffin.

### Histology and immunohistochemistry

Four micrometre sections were cut from paraffin-embedded blocks of tumours and organs, and mounted on slides. Dewaxed and hydrated sections were stained in Harris haematoxylin solution (Thermo Scientific) and blued with Scott’s tap water substitute (Sigma-Aldrich). Sections were then stained in eosin solution (Sigma-Aldrich), dehydrated and mounted in DPX (VWR International, Lutterworth, UK). For immunohistochemical analyses, freshly cut tissue sections were stained by primary antibodies against carcinoma embryonic antigen (II-7), vimentin (V9) and cytokeratin (AE1-AE3), using Dako Autostainer Link 48 on an automated staining platform and the Dako EnVision FLEX detection kit (Dako, Ely, UK). Slides were counterstained with haematoxylin before dehydration and mounting in DPX (VWR International).

### Digital microscopy

Photographs of live cultures were taken using a Leica DM IRBE inverted microscope (Leica Microsystems, Milton Keynes, UK) with a Hamamatsu ORCA-ER camera supported with OpenLab 3.1.7 (Improvision, Convetry, UK), or using a LumaScope 500 inverted microscope (Etaluma, Labtech, Uckfield, UK). For confocal immunofluorescence microscopy, CSC-like cells and non-CSCs were grown in Nunc Lab-Tek cover-slip chamber slides to subconfluency and fixed with acetone/methanol for staining with a series of primary mAbs against α-SMA (1A4), N-cadherin (8C11), cytokeratin-14 (LL001), CK-18 (RGE53), extra domain A-fibronectin (IST-9) and vimentin (V9) (all from Santa Cruz Biotechnology, Heidelberg, Germany), together with appropriate isotype controls, followed by AF488-conjugated secondary antibodies with counterstaining for cell nucleus by DAPI. Prepared slides were imaged and analysed using a Zeiss AxioVert fluorescence microscope (Zeiss, Cambridge, UK). Images were processed with Photoshop 6.0 (Adobe, San Jose, CA, USA). For video microscopy, target cells were incubated overnight in Ibidi chamber slides (Martinsried, Germany) coated with fibronectin (Millipore). For intracellular Ca^2+^ measurements, CD8^+^ T cells were loaded with 1 μm Fura-2/AM (Invitrogen) and analysed using a DMI 6000B microscope (Leica Microsystems). Cells were illuminated every 10 s with a 300 W xenon lamp by using 340/10 nm and 380/10 nm excitation filters. Emission at 510 nm was captured using a Cool Snap HQ2 camera (Roper, Tucson, AZ, USA) with Metafluor software (Molecular Devices, Sunnyvale, CA, USA). Ratio measurements were performed with Imaris 8.1 imaging software (Oxford Instruments, Abingdon, UK).

### Statistics

Data were analysed using two-tailed Student’s *t*-tests for normally distributed data and Mann–Whitney tests for non-parametric data (GraphPad Prism 6.0, La Jolla, CA, USA). Paired data were analysed using Wilcoxon matched-pairs signed-rank tests. Differences between groups were analysed using one-way analysis of variance with Bonferroni’s post tests; two-way analysis of variance was used when comparing groups with independent variables. Differences were considered significant as indicated in the figures: *P<0.05; **P<0.01; ***P<0.001.
