# Supplementary material for: Synergistic targeting of breast cancer stem-like cells by human γδ T cells and CD8+ T cells
Source: Immunol Cell Biol. 2017 May 9;95(7):620–9. doi: 10.1038/icb.2017.21 (PMC5550559; doi:10.1038/icb.2017.21)
Supplement: Supplementary Information [file icb201721x1.docx]

**Synergistic targeting of breast cancer stem-like cells by human γδ T cells and CD8^+^ T cells**

Hung-Chang Chen^1,*^, Noémie Joalland^2,3^, John S. Bridgeman^1,‡^, Fouad S. Alchami^4^, Ulrich Jarry^2,3^, Mohd Wajid A. Khan^1^, Luke Piggott^1,5^, Yasmin Shanneik^1^, Jianqiang Li^6^, Marco J. Herold^6,7^, Thomas Herrmann^6^, David A. Price^1,8^, Awen M. Gallimore^1,8^, Richard W. Clarkson^5,9^ Emmanuel Scotet^2,3^, Bernhard Moser^1,8^, and Matthias Eberl^1,8^

**SUPPLEMENTARY MATERIAL**

| **Table S1. Basic histological examination of tumours derived from CSC-like cells or non-CSCs in NSG mice** | | | | | | | | |
| --- | --- | --- | --- | --- | --- | --- | --- | --- |
| Cell type and dose (cells/mouse) | Mouse no. | Mouse mammary tissue | Degree of differentiation | Possible squamous differentiation | Inter-cellular bridges | Necrosis | Apoptosis | Days that tumour size reached 1 cm diameter |
| 10^3^ CSC-like cells  per mouse | 1 | N | Poor | Y | 0 | None | 0 | 112 |
|  | 2 | Y | Poor | N | 0 | Moderate | 1 | 95 |
|  | 3 | Y | Poor | N | 0 | None | 0 | 195 |
|  | 4 | Y | Poor | N | 0 | None | 0 | 147 |
|  | 5* | - | - | - | - | - | - | 130 |
|  | 6 | N | Poor | N | 0 | Mild | 0^♯^ | 144 |
| 2×10^6^ CSC-like cells  per mouse | 1 | Y | Poor | N | 0 | None | 0 | 51 |
|  | 2 | N | Poor | N | 0 | Extensive | 1 | 53 |
|  | 3 | Y | Poor | N | 0 | Moderate | 1 | 63 |
|  | 4 | Y | Poor | Y | Possible | Mild | 0 | 46 |
|  | 5 | Y | Poor | N | 0 | None | 0 | 60 |
|  | 6 | N | Poor | N | 0 | None | 0 | 56 |
| 2×10^6^ non-CSCs  per mouse | 1 | N | Well | Y | Present | None | 0 | 91 |
| *Tumour collected from this mouse was only analyzed by flow cytometry and was not included in histological examinations.  ^♯^Apoptosis noticed in infiltrated neutrophils. | | | | | | | | |

| **Table S1. Basic histological examination of tumours derived from CSC-like cells or non-CSCs in NSG mice (continued)** | | | | | | | | | |
| --- | --- | --- | --- | --- | --- | --- | --- | --- | --- |
| Cell type and dose (cells/mouse) | Mouse no. | Cytoplasmic vacuoles | Multi-nucleate giant cells | Bizarre nuclei | Large nucleus | Intra-nuclear inclusion | Separate vascular proliferation | Inter-cellular bridges | Neutrophil infiltration |
| 10^3^ CSC-like cells  per mouse | 1 | 1 | 0 | 0 | 1 | 0 | 0 | 0 | 1 |
|  | 2 | 1 | 1 | 1 | 1 | 1 | 1 | 0 | 3 |
|  | 3 | 1 | 1 | 1 | 1 | 1 | 0 | 0 | 2 |
|  | 4 | 0 | 0 | 0 | 1 | 1 | 0 | 0 | 2 |
|  | 5* | – | – | – | – | – | – | – | – |
|  | 6 | 1 | 1 | 1 | 1 | 1 | 1 | 0 | 2 |
| 2×10^6^ CSC-like cells  per mouse | 1 | 0 | 1 | 1 | 1 | 0 | 0 | 0 | 1 |
|  | 2 | 0 | 1 | 1 | 1 | 0 | 0 | 0 | 1 |
|  | 3 | 0 | 1 | 1 | 1 | 0 | 0 | 0 | 1 |
|  | 4 | 1 | 0 | 0 | 1 | 0 | 0 | Possible | 1 |
|  | 5 | 0 | 0 | 0 | 0 | 0 | 0 | 0 | 0 |
|  | 6 | 0 | 0 | 0 | 1 | 0 | 0 | 0 | 0 |
| 2×10^6^ non-CSCs  per mouse | 1 | 0 | 0 | 0 | 0 | 0 | 0 | Present | 0 |
| *Tumour collected from this mouse was only analyzed by flow cytometry and was not included in histological examinations. | | | | | | | | | |

| **Table S2. Epithelial differentiation of tumours derived from CSC-like cells and non-CSCs in NSG mice** | | | | | | | | | | |
| --- | --- | --- | --- | --- | --- | --- | --- | --- | --- | --- |
| Cell type and dose (cells/mouse) | Mouse no. | % CD44^hi^ CD24^lo^ cells | % CD44^lo^ CD24^hi^ cells | % Tumour tissue with epithelioid appearance | Mitosis rate^♯^ of epithelial-like component | Mitosis rate^♯^ of mesenchymal-like component | AE1/AE3 IHC score^§^ | % AE1/AE3^+^ stain of whole tissue | Vimentin IHC score | % Vimentin^+^ stain of whole tissue |
| 10^3^ CSC-like cells  per mouse | 1 | 40.4 | 7.98 | 25 | 44 | 120 | 1 | 25 | 3 | 75 |
|  | 2 | 54.2 | 8.8 | 80 | 57 | 21 | 2 | 80 | 3 | 20 |
|  | 3 | 0.847 | 15.6 | 85 | 42 | 33 | 1 | 5 | 3 | 95 |
|  | 4 | 2.11 | 56.3 | 10 | N/A | 22 | 1 | 5 | 3 | 95 |
|  | 5* | 26 | 64.2 | – | – | – | – | – | – | – |
|  | 6 | 0.54 | 19.9 | 100 | 113 | N/A | 2 | 75 | 3 | 25 |
| 2×10^6^ CSC-like cells  per mouse | 1 | 56.6 | 4.18 | 100 | 68 | N/A | 1 | 10 | 3 | 90 |
|  | 2 | 9.6 | 28.3 | 100 | 21 | N/A | 2 | 20 | 3 | 80 |
|  | 3 | 46.3 | 9.24 | 100 | 70 | N/A | 1 | 5 | 3 | 95 |
|  | 4 | 15.3 | 15.4 | 100 | 59 | N/A | 0 | 0 | 3 | 100 |
|  | 5 | 30.9 | 23.8 | 1 | N/A | 39 | 0 | 0 | 3 | 100 |
|  | 6 | 20 | 18.4 | 50 | 35 | 35 | 1 | 20 | 3 | 80 |
| 2×10^6^ non-CSCs  per mouse | 1 | 0.024 | 96.4 | 100 | 20 | N/A | 3 | 100 | 1 | 0 |
| *Tumour collected from this mouse was only analyzed by flow cytometry and was not included in histological examinations.  ^♯^Mitotic rate: mitosis per 10 high power fields (0.5 mm in diameter) in the epitheliod or mesenchymal component; N/A is applied if the area is smaller than 0.5 mm in diameter.  ^§^IHC score: strength of the staining for indicated marker | | | | | | | | | | |

**Figure S1. Isolation of CD44^hi^ CD24^lo^ CSC-like cells and CD44^lo^ CD24^hi^ non-CSCs from the parental HMLER cell line.** (***A***) HMLER cells were labelled with anti-CD44 and anti-CD24 mAbs and sorted to purities >99% using a BD FACS Aria cell sorter. (***B***) CD44/CD24 expression profiles of the sorted cell populations after the sort and (***B***) after 32 days of culture under normal adherent culture conditions. (***C***) GD2 expression of CSC-like cells and non-CSCs maintained in normal adherent culture as assessed by flow cytometry.

**Figure S2. Generation of CD44^hi^ CD24^lo^ CSC-like cells and CD44^lo^ CD24^hi^ non-CSCs co-expressing tdTomato and FluM1.** (***A***) CSC-like cells and non-CSCs were transduced with lentiviral particles delivering a bicistronic tdTomato-T2A-M1 gene cassette. Successfully transduced tdTomato^hi^ cells were sorted to purities >99% using a BD FACS Aria cell sorter. (***B***) tdTomato expression and CD44/CD24 phenotype of transduced CSC-like cells and non-CSCs as assessed by flow cytometry. (***C***) Intracellular expression of FluM1 by transduced CSC-like cells and non-CSCs as assessed by flow cytometry.

**Figure S3. Development of tumours derived from CSC-like cells and non-CSCs co-expressing tdTomato and FluM1.** CSC-like cells or non-CSCs were xenotransplanted with matrigel into the mammary fat pad of NSG mice at two different doses; at a high dose of 2 × 10^6^ cells/mouse and at a low dose of 1 × 10^3^ cells/mouse (*n*=6 per group). Tumour development and growth were monitored (***A***) by live imaging of tdTomato using the Kodak Fx-Pro system and (***B***) by caliper measurements.

**Figure S4. Increased resistance of CSC-like cells to MHC-restricted cytotoxic CD8**^+^ **T cells.** CSC-like cells and non-CSCs were pulsed with FluM1 p58-66 peptides or CMV pp65 p495-503 peptides, labelled with CellVue or PKH26, and mixed in equal numbers to generate the different combinations as shown in the figure. These combinations were then used as targets for killing by (***A***) FluM1 specific CD8^+^ T cells, or (***B***) CMV pp65-specific CD8^+^ T cells, at different effector:target (E:T) ratios. Specific killing of CellVue and PKH26-labelled target cells was assessed by live/dead staining and analysed by flow cytometry. Data shown are representative of two independent experiments performed in duplicate.

**Figure S5. Sensitisation of CSC-like cells and non-CSCs to γδ T cell-mediated killing by FPPS knockdown.** CSC-like cells and non-CSCs transduced with FUTG-INSR control vector or FUTG-SR22 vector were pr-treated overnight with 0.1 μg/ml doxycycline to induce expression of shRNA targeting FPPS in cells with FUTG-SR22 expression. After overnight treatment, CSC-like cells and non-CSCs transduced with FUTG-INSR or FUTG-SR22 vector were mixed in equal numbers and used as targets for γδ T cell-mediated killing, at different effector:target (E:T) ratios. Specific killing of CellVue and PKH26-labelled target cells was assessed by live/dead staining and analysed by flow cytometry. Data shown are from one experiment performed in duplicate.

**Movie S1. Killing of CSC-like cells by antigen-specific CD8^+^ T cells.** FluM1-specific CD8^+^ T cells were loaded with Fura2 and added to FluM1-transduced target cells. The video shows the kinetics of intracellular Ca^2+^ levels and tumour cell killing by CD8^+^ T cells, and is representative of four experiments performed. Cells were illuminated every 10 seconds, videos were recorded with a time lapse of 12 images per second.

**Movie S2. Sensitisation of CSC-like cells to killing by antigen-specific CD8^+^ T cells using IFN-γ.** FluM1-specific CD8^+^ T cells were loaded with Fura2 and added to FluM1-transduced target cells that had been pre-treated with 100 U/ml IFN-γ for 24 hours. The video shows the kinetics of intracellular Ca^2+^ levels and tumour cell killing by CD8^+^ T cells, and is representative of four experiments performed. Cells were illuminated every 10 seconds, videos were recorded with a time lapse of 12 images per second.

**Movie S3. Sensitisation of CSC-like cells to killing by antigen-specific CD8^+^ T cells using Vγ9/Vδ2 T cells.** FluM1-specific CD8^+^ T cells were loaded with Fura2 and added to FluM1-transduced target cells that had been pre-treated with γδ T cell conditioned medium for 24 hours. The video shows the kinetics of intracellular Ca^2+^ levels and tumour cell killing by CD8^+^ T cells, and is representative of four experiments performed using γδ T cells from three different donors. Cells were illuminated every 10 seconds, videos were recorded with a time lapse of 12 images per second.
